# How much risk does delirium represent for the development of dementia?: Retrospective cohort study from over 260,000 patients record in a solitary institution

**DOI:** 10.3389/fpsyt.2024.1387615

**Published:** 2024-09-13

**Authors:** Hironari Minami, Katsunori Toyoda, Takeo Hata, Masami Nishihara, Masashi Neo, Keiichiro Nishida, Tetsufumi Kanazawa

**Affiliations:** ^1^ Department of Neuropsychiatry, Faculty of Medicine, Osaka Medical and Pharmaceutical University, Takatsuki, Osaka, Japan; ^2^ Department of Pharmacy, Osaka Medical and Pharmaceutical University Hospital, Takatsuki, Osaka, Japan

**Keywords:** delirium, dementia, cognitive decline, retrospective cohort study, subsequent dementia onset

## Abstract

**Background:**

Delirium frequently affects the consciousness of the elderly, particularly those in hospitals. Evidence increasingly associates linking delirium history to an increased risk of dementia. However, most studies are limited in scope, focusing mainly on postoperative or intensive care units with small patient samples, which affects the broader applicability of their findings.

**Aims:**

To elucidate the precise incidence of delirium and the subsequent onset of dementia within whole inpatients. Additionally, we aimed to explore the correlation between the emergence of delirium during hospitalization and the subsequent manifestation of dementia.

**Design, setting, and participants:**

We conducted a retrospective cohort analysis employing a decade-long electronic medical record dataset consisted of 261,123 patients in Osaka Medical and Pharmaceutical University Hospital. Key analyses were performed October 2022 to January 2023.

**Main outcomes and measures:**

The primary outcome, dementia onset, was determined by prescriptions for the anti-dementia drugs donepezil, galantamine, memantine, or rivastigmine, which are approved for use in Japan.

**Results:**

10,781 patients met the inclusion criteria. The median interval between the onset of dementia was 972.5 days for individuals without a history of delirium, whereas for those with a history of delirium, it was notably shorter at 592.5 days. This disparity culminated in a hazard ratio of 5.29 (95% confidence interval: 1.35-20.75) for subsequent dementia onset.

**Conclusions and relevance:**

This investigation underscores the imperative significance of directing attention toward preventive measures against delirium during hospitalization, alongside the necessity of diligent monitoring and intervention for cognitive decline in patients who encounter delirium.

## Introduction

In contemporary times, dementia has surged as a prevalent ailment in both psychiatric and internal medical and surgical domains. Projections indicate an anticipated surge in dementia cases from 57.4 million globally in 2019 to 152.8 million by 2050 (1). The impact of dementia is consistently reflected in WHO disability-adjusted life years, burden on families and caregivers (2) and broader societal economic costs (3). It is universally acknowledged that dementia’s etiology encompasses diverse lifestyle habits and ailments that induce neurofibrillary and inflammatory transformations in neurons. Among internal conditions, hypertension (4), diabetes mellitus (5), and dyslipidemia (6) stand as established examples. In terms of psychiatric disorders, depression and bipolar affective disorder increase the risk of developing dementia with each severe episode that requires hospitalization (7).

On the other hand, delirium is also a common disease that is frequently seen in daily medical care. Delirium is a disturbance of consciousness that is mainly caused by a sudden decline or change in cognitive functions such as attention, comprehension, and memory, and is especially common in hospitalized elderly patients who are in poor general condition or have undergone a major external invasion such as surgery. A meta-analysis of 33 studies published in 2020 found an overall prevalence of delirium as high as 23% in elderly hospitalized patients (8).

The longstanding notion that a history of delirium serves as a harbinger for dementia’s emergence is well-established, with multiple reports corroborating delirium’s role as a precursor to dementia. For example,a prior UK study in 1999 revealed a yearly dementia incidence of 5.6% over a span of 3 years in the non-delirium group, contrasting with an 18.1% annual incidence in the delirium-afflicted cohort. This yielded an unadjusted relative risk of dementia amounting to 3.23 (95% confidence interval: 1.86-5.63) within the delirium-affected group (9). In a substantial decade-long prospective analysis encompassing individuals aged 85 years and above, the presence of delirium was quantified to yield an odds ratio of 8.7 (95% confidence interval: 2.1-35) for the development of subsequent dementia (10). Further strengthening this connection, a meta-analysis involving two reports yielded a higher value of 12.5, albeit from a more limited study involving 35 cases of incident dementia (11). A recent meta-analysis reported a Hedges’ g value of 0.45 (12), underscoring that this metric remains variable. The hazard ratio for mortality within a year due to delirium alone stands at 1.6; however, when delirium is coupled with dementia, this ratio escalates to 2.3 (13). Nonetheless, it is essential to note that several of these prior investigations grapple with limitations stemming from modest sample sizes or a focus on specific contexts like postoperative or ICU-related delirium. While these studies boast robust internal validity, their external validity is constrained. It is not surprising that many studies have focused on postoperative or ICU patients, since studies in settings with low rates of delirium are inevitably limited.

## Objectives

This study’s core intent is to elucidate the true incidence of delirium and dementia within a singular institution and probe the extent to which delirium during hospitalization serves as a risk determinant for subsequent dementia across a broader patient cohort, transcending postoperative or ICU-related cases.

## Materials and methods

### Data source

In this study, we used electronic medical record data and incident report data from Osaka Medical and Pharmaceutical University Hospital for a 10-year period from 2012 to 2021. The hospital’s incident report data includes a variety of incidents that occurred in the hospital, including near-misses, errors, mistakes, and accidents, regardless of their impact on patients. The hospital is a university hospital and provides medical services as a general hospital. It has 903 beds and 31 clinical departments and is located in Takatsuki City, Osaka Prefecture. The medical area is the Hokusetsu region, with a population of approximately 1.65 million.

### Ethical consideration

This study was conducted in accordance with the Declaration of Helsinki and approved by the ethics committee of Osaka Medical and Pharmaceutical University. The approval number is 2022-169. Since this is a retrospective observational study without intervention or invasion, the requirement for informed consent was waived.

### Study design

A retrospective cohort study was conducted to examine the impact of the onset of delirium on the risk of developing subsequent dementia (**Supplementary Figure 1**). This study was conducted according to the STROBE statement (14). Exposure was defined as delirium onset and outcome as dementia onset. The cohort entry date was the date of first delirium onset for the exposed group and the date of hospital admission for the non-exposed group. The cohort entry date for the non-exposed group was the date of hospitalization because all delirium in this study occurred during hospitalization. The following exclusion criteria were applied to outpatients or inpatients at Osaka Medical and Pharmaceutical University Hospital during the inclusion period: patients with no medical history for at least one year prior to the cohort entry date, patients who developed delirium or dementia prior to the cohort entry date, patients with a time interval of less than 180 days from cohort entry date to last clinic visit (i.e., follow-up period), patients with a time between cohort entry date and dementia onset of less than 180 days, patients with missing covariates, patients younger than 65 years of age. Patients were followed up from 180 days after the cohort entry date until the first of the following three events: onset of dementia, disenrollment, or end of study period.

### Exposure and outcomes

The exposure factor, onset of delirium, was identified by consultation orders for delirium treatment issued by the primary department to the psychiatry department during hospitalization. The outcome, onset of dementia, was identified by prescription of the anti-dementia medications donepezil, galantamine, memantine, or rivastigmine. Currently, these are the only four anti-dementia drugs approved in Japan.

### Covariates

Fall history was obtained from incident report data, and other variables were obtained from electronic medical record data. Among the variables, age and sex were determined at the cohort entry date. Variables such as height, weight, comorbidities, department, drug allergies, dialysis, cancer chemotherapy, history of hospitalization, history of surgery, laboratory values, medications, family, smoking history, alcohol consumption, and history of falls were obtained for the 365 days prior to the cohort entry date (baseline period).

Comorbidities are identified by International Statistical Classification of Diseases and Related Health Problems, 10th revision (ICD-10) codes (**Supplementary Table 1**). Drugs are identified by Anatomical Therapeutic and Chemical (ATC) classification system (**Supplementary Table 2**).

Benzodiazepine or antipsychotics doses (mg/day) were converted according to diazepam or chlorpromazine equivalence (15).

### Statistical analysis

Variables and their distributions were described by univariate and bivariate analyses, and the two groups were compared using Fisher’s exact test for categorical variables and Wilcoxon rank-sum test for continuous variables, respectively.

In a multivariate logistic regression model, the acquired covariates were used to calculate the propensity score of the patients. Then, because the presence or absence of delirium is nonrandom, propensity score matching was used to compare between groups with similar distributions of measured covariates. The propensity score matched pairs were created one-to-one by nearest-neighbor matching with sampling without replacement and a caliper of 0.2. After matching, covariate balance was evaluated by Fisher’s exact test or Wilcoxon rank-sum test and standardized mean difference. If the *p*-value was less than 0.05 and the standardized mean difference was greater than 10%, the covariate was considered unbalanced between the two groups and was included in the final model. Kaplan-Meier curves were constructed based on the follow-up period of each patient, and differences between the two groups were compared by log-rank test. Finally, stratified multivariate Cox regression analysis was used to estimate the effect of delirium on the hazard of dementia.

Variance inflation factors (VIFs) ≥10 were considered evidence of multicollinearity. All *p*-values were reported using two-tailed tests, and the significance level was set at 5%. Analyses were performed using R version 4.2.2 (R Development Core Team, Vienna, Austria).

### Sensitivity analysis

Since propensity score matching is performed randomly, the value of the obtained hazard ratio is affected by the pairs created. Therefore, the process of creating propensity score matched pairs and calculating the hazard ratio of delirium to dementia by stratified univariate Cox regression analysis was repeated up to 100,000 times using the Monte Carlo method, and the distribution of the obtained hazard ratios was examined.

To assess the impact of unmeasured confounding in this study, the E-value of the hazard ratio was calculated as a quantitative bias analysis. The E-value is defined as the minimum strength of unmeasured confounding to negate the observed results. A higher E-value means that unmeasured confounding must be strong to overturn the observed association (16).

We then performed conventional Cox regression analysis without propensity scores. At first, univariate Cox regression analysis was used to estimate the hazard ratio for developing dementia in patients with and without delirium. Patients were censored at disenrollment or end of study period. Proportional hazard was confirmed by the proportional hazard test by Schoenfeld residuals. In addition, multivariate Cox regression models adjusted for covariates with *p*<0.05 in univariate Cox regression analysis were used to estimate the hazard ratios for developing dementia in patients with and without delirium.

Hazard ratios for developing dementia were estimated by a multivariate Cox regression model using the propensity score as a covariate.

In the main analysis, the study excluded patients who developed dementia within 180 days of the onset of delirium. This period was extended to 365 days for the propensity score matching and stratified Cox regression analysis.

## Results

### Characteristics of study patients

**Figure 1:**
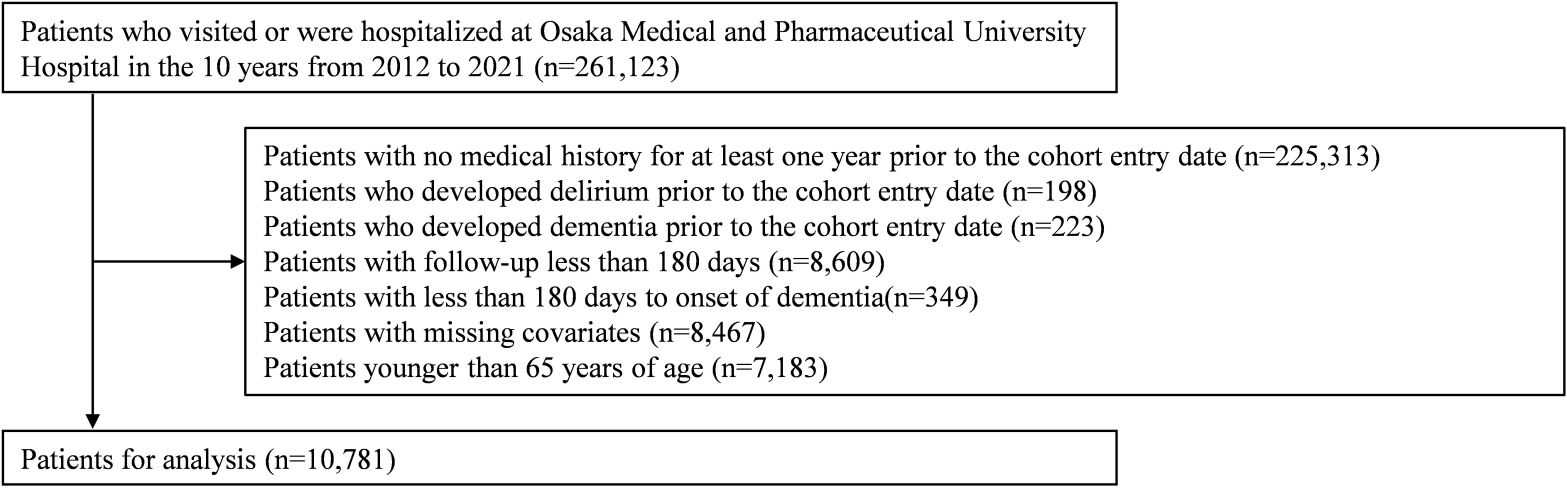
Patient selection flow chart.

Of all 261,123 patients who visited Osaka Medical and Pharmaceutical University Hospital during the 10-year period from 2012 to 2021, the final number of eligible patients after applying the exclusion criteria was 10,781 (**Figure 1**).

**Supplementary Table 3** shows the background of the target patients. The median age of the 10,781 study patients was 74 years, and 45.1% were female. Malignant neoplasms (27.2%), diabetes mellitus (14.8%), and hypertension (9.4%) were common as comorbidities, and cardiology (31.6%), gastroenterology (28.4%), and urology (22.3%) were common among clinical departments. Of the 10,781 patients studied, 1.4% had dialysis, 6.5% had chemotherapy, and 48.8% had all-packaged oral medications, with a median of 5 medications being taken. Antithrombotic drugs (31.1%), non-steroidal anti-inflammatory drugs (NSAIDs) (30.8%), and benzodiazepine (14.9%) were the most commonly used drugs. A history of falls was seen in 0.7% of patients. Of the 10,781 eligible patients, 582 were in the delirium group and 10,199 in the non-delirium group. The overall incidence of dementia was 2.2%, higher in the delirium group (5.5% vs. 2.0%). The time to onset of dementia was shorter in the delirium group than in the non-delirium group (median 592.5 days vs. 972.5 days). The delirium group was older and had higher rates of most comorbidities, departments, dialysis, packaging, medications, and history of falls.

The patient background after propensity score matching is shown in **Supplementary Table 4**. There were 288 patients in both the delirium and non-delirium groups, with a good balance between the two groups except for the number of drugs and falls, where *p*<0.05 and standardized mean difference (SMD)>0.1.

### Estimating the hazard ratio of delirium to dementia

**Figure 2:**
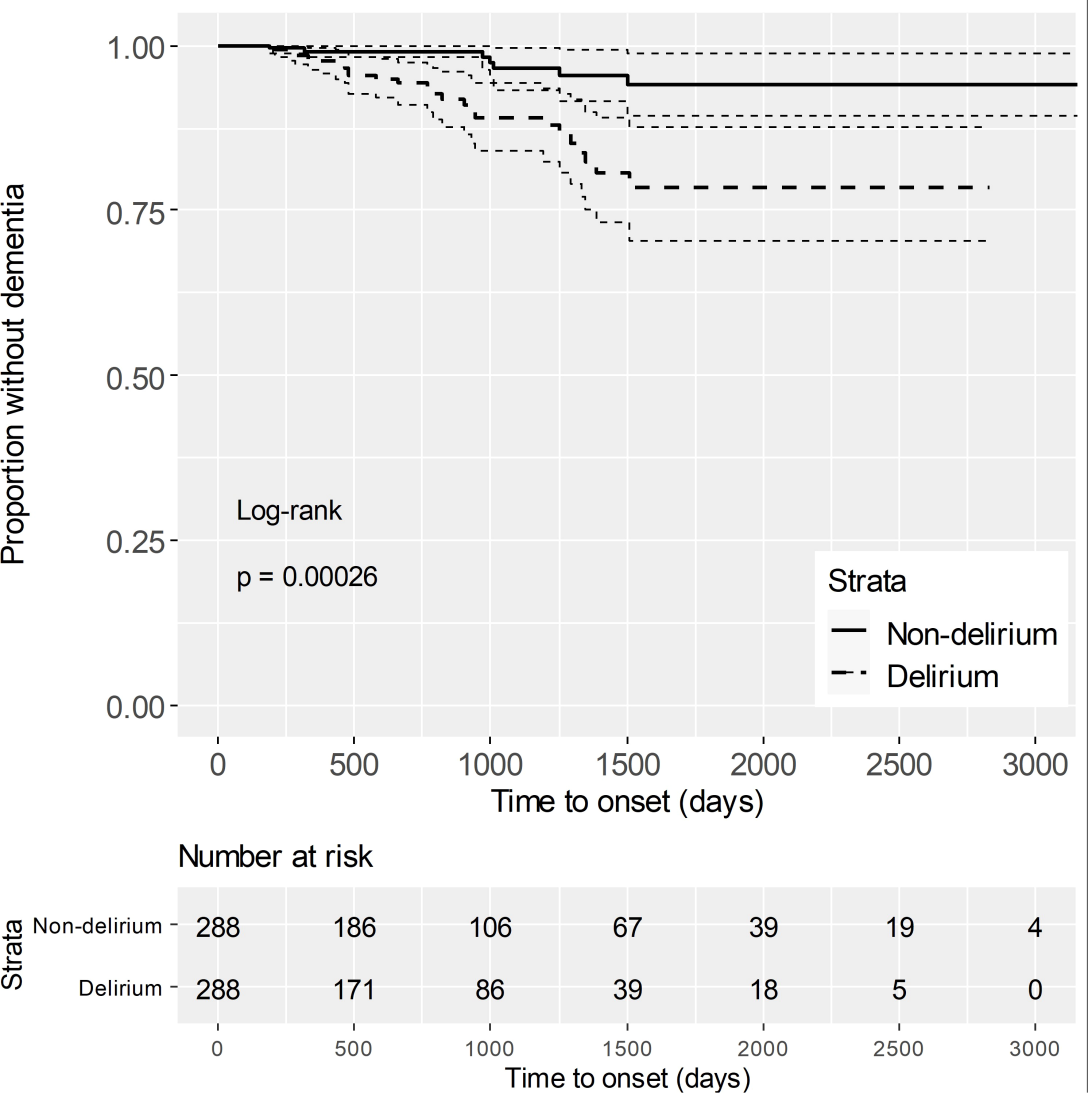
Kaplan-Meier curves after propensity score matching The solid line represents the survival rate (proportion without dementia) for the non-delirium group. The dashed line represents the survival rate for the delirium group. Dotted lines indicate the 95% confidence intervals, reflecting the uncertainty in survival estimates. The "Number at risk" below the plot shows the number of individuals at risk for dementia at each time point. The log-rank test yielded a p-value of 0.00026, indicating a significant difference in dementia risk between the two groups.

After propensity score matching, the risk of dementia was compared between the delirium group and the non-delirium group using the log-rank test, and the risk was significantly higher in the delirium group (**Figure 2**). Dementia risk due to delirium was analyzed by stratified univariate Cox regression analysis and the hazard ratio (HR) was 3.75 (1.24–11.30) (Model 1 in **Table 1**). Furthermore, a stratified multivariate Cox regression analysis was performed by adding the unbalanced number of drugs and fall as explanatory variables after propensity score matching, and the HR was 5.29 (1.35–20.75) (Model 2 in **Table 1**).

**Table 1:** Stratified univariate Cox regression analysis to estimate the hazard ratios for developing dementia after propensity score matching.

Variable	HR	95% CI	*p*-value	VIF
Model 1				
Delirium	3.75	1.24-11.30	0.019^*^	
Model 2				
Delirium	5.29	1.35-20.75	0.017^*^	1.38
Number of drug	0.97	0.90-1.04	0.380	1.21
Fall	0.26	0.01-5.65	0.391	1.22

CI, confidence interval; HR, hazard ratio; VIF, variance inflation factor. ^*^p < 0.05.

### Sensitivity analysis

Since propensity score matching is performed randomly, the process from propensity score matching to calculating the hazard ratio of delirium to dementia by stratified univariate Cox regression analysis was repeated up to 100,000 times using the Monte Carlo method. We investigated what kind of distribution it shows. The average hazard ratio of 100,000 samples was 8.22 (**Table 2**).

**Table 2:** Estimation of hazard ratios by Monte Carlo method with repeated propensity score matching.

n	Mean	SD	SE	95% CI
10	7.41	5.40	1.71	3.55–11.27
100	7.82	3.93	0.39	7.04–8.60
1,000	8.15	4.32	0.14	7.88–8.41
10,000	8.27	4.25	0.04	8.18–8.35
100,000	8.22	4.23	0.01	8.19–8.24

CI, confidence interval; SD, standard deviation; SE, standard error.

Quantitative bias analysis was performed to examine the effects of unmeasured confounding. The E value for the hazard ratio of model 2 in **Table 1** was 10.05.

Univariate Cox regression analysis was performed in 10,781 target patients before propensity score matching, and variables with statistically significant association with the onset of dementia were entered into multivariate Cox regression analysis. However, variables for which proportional hazards did not hold, and height, weight, and leukocyte were excluded to avoid multicollinearity. As a result, the hazard ratio of delirium to dementia was 2.90 (1.80–4.68) (**Supplementary Table 5**). Also, the hazard ratio obtained by the Cox regression model adjusted by propensity score only was 6.48 (3.65–11.48) (**Supplementary Table 6**).

We extended the exclusion period from 180 days to 365 days following the onset of delirium and conducted an additional analysis using the same method. As a result, the stratified multivariable Cox regression analysis after propensity score matching showed a hazard ratio of 5.00 (1.10–22.8) for the impact of delirium on the onset of dementia.

## Discussion

### Outline

This study constitutes a component within a series of inquiries harnessing extensive data derived from a decade-long observation at Osaka Medical and Pharmaceutical University Hospital. As previously elucidated, the outcomes of this investigation consistently align in the same direction, unequivocally substantiating that the inception of delirium during hospitalization starkly augments the subsequent vulnerability to dementia. Variations in statistical methodologies notwithstanding, the hazard ratio for dementia development subsequent to delirium escalates significantly to 5.29 (95% confidence interval: 1.35-20.75). Moreover, the temporal pattern discerned indicates that the median duration to dementia emergence within the delirium cohort is 592.5 days, in contrast to the extended interval of 972.5 days observed in the non-delirium cohort.

### Delirium: predictor or causative agent of dementia?

The precise nature of delirium’s role—whether it functions solely as a predictor for future dementia development or potentially serves as a proximate causative trigger for dementia—remained unclarified by this study. The existing body of knowledge, gleaned from earlier investigations unequivocally establishes delirium as a predictive factor for dementia (9, 10, 12). At the same time, the existence of common underlying physiological and pathological mechanisms, such as inflammatory changes, oxidative stress changes, and neuronal dysfunction, as well as the existence of common biomarkers, are also considered to be possible (17) and common biomarkers (18).

However, within clinical practice, instances are observed where patients with recurrent episodes of postoperative delirium, for instance, exhibit expedited cognitive decline, often culminating in eventual dementia. In this regard, some reports suggest that postoperative delirium itself does not contribute to the development of dementia, but rather the cascade of inflammatory cytokines in the central nervous system associated with surgical invasion (19). A 2020 meta-analysis emphasizes that postoperative delirium may not directly act as a risk factor for cognitive decline but could rather manifest as an epiphenomenon reflecting preoperative cognitive impairment, gradually being acknowledged as a mere biomarker of existing cognitive degradation (12). However, a study of approximately 40,000 patients aged 50 years or older in 2022 who underwent surgical treatment requiring hospitalization noted that the odds ratio for developing dementia within one year of developing postoperative delirium increased to 13.9 (95% confidence interval 12.2-15.7) (20). This suggests that postoperative delirium itself may be a strong factor influencing the development of dementia. Additionally, indications exist of a dose-response correlation between the severity of postoperative delirium and subsequent long-term cognitive decline (21).

Fundamentally, the query persists whether delirium encountered during general ward hospitalization can be equated with postoperative delirium subsequent to surgical procedures or delirium manifesting in an intensive care unit (ICU) setting. Delirium development is governed by three distinct factors: preparatory factors, inducers, and direct factors. The relative impact of these factors is likely to vary between cases of delirium occurring during general ward hospitalization compared to cases emerging post-surgery or in the ICU. The former scenario would place greater emphasis on preparatory factors like inherent brain function vulnerability, thereby rendering delirium onset more indicative of future dementia development. In contrast, the latter cases would accentuate the influence of direct factors such as surgical trauma and critical deterioration of overall health status, potentially correlating more directly with dementia initiation. While disentangling the individual contributions of these factors to dementia onset warrants separate investigation, it remains broadly recognized that delirium serves as both a predictor of dementia initiation and a condition that occasionally expedites cognitive regression, thus exerting a direct impact on the onset and progression of dementia (22, 23).

The results of our analysis suggest that both the number of drugs and falls may also be significant risk factors associated with the onset of dementia. Therefore, we conducted a stratified multivariable Cox regression analysis by including these two factors as covariates. The results showed that the hazard ratio for delirium increased from 3.75 (1.24–11.30) in the stratified univariable Cox regression analysis to 5.29 (1.35–20.75). This further demonstrates that delirium is a clearly independent risk factor for the future onset of dementia. Regarding the inclusion of the number of drugs as an unbalanced covariate, as mentioned in the Introduction, various internal medical conditions and psychiatric disorders are widely known to be risk factors for the onset of dementia. Therefore, it is reasonable to consider that the risk of developing dementia increases when pharmacotherapy is administered for comorbid physical and mental conditions. On the other hand, regarding falls, they can be interpreted as an indicator of lower limb motor function decline. Meta-analyses have shown that the risk of developing dementia increases as balance function and lower limb motor function decline (24).

In this study, we optimized the analysis by using the Monte Carlo method, repeatedly performing up to 100,000 iterations of the stratified univariable Cox regression process based on similar propensity score matching. The resulting distribution of hazard ratios showed a peak around 8.27 after 10,000 iterations. This suggests that the actual hazard ratio might be slightly higher than the result obtained from the stratified multivariable Cox regression analysis, which was 5.29. Therefore, it is possible that the true hazard ratio could be higher. Future studies using larger datasets with more dementia onset outcomes are warranted to perform similar analyses.

### Strengths and limitations

The strengths of previous investigations have consistently highlighted the association between delirium and heightened dementia susceptibility, often accompanied by elevated mortality rates in affected patients. However, the majority of these studies grapple with restricted sample sizes (25–27) or have focused only on postoperative delirium (28–32) or delirium in the ICU (33–35). In contrast, the present analysis demonstrates a distinctive strength by being conducted within a single institution, leveraging an ample and robust sample size. Previous studies have reported that the incidence of delirium in the study period ranged from around 20% to 50%, and some have reported that the incidence of delirium increases to 80% or more in the ICU setting (36). In comparison, the incidence of delirium in our study, encompassing a broad spectrum of patients admitted to general hospital wards, stands at 5.4% (582 out of 10781 patients), thus diverging from previous studies. This deviation can be attributed to the comprehensive nature of our cohort, encompassing a wider patient demographic, thereby endowing the study with enhanced statistical power for the meticulous adjustment of numerous covariates that potentially underpin dementia development (35). Some previous studies have also used screening tools for dementia, such as the MMSE, to assess whether or not dementia has developed, but it has been noted that such cognitive tests have limitations, such as ceiling effects, practice effects due to repeated assessment, and difficulty in assessing subtle cognitive functions (37). In addition, not all patients in a retrospective observational study such as the present study had the MMSE. Therefore, in this study, the outcome of developing dementia was detected in the form of the first dose of anti-dementia medication administered by a psychiatrist.

However, our study has several limitations: Firstly, the identification of delirium within our study hinged on the keyword “delirium” within electronic medical records, introducing potential variability in diagnostic criteria as they were reliant on clinicians’ judgment across different departments. Thus, the uniformity of delirium severity remains uncertain. Secondly, the criterion for dementia was established based on the presence of anti-dementia medication. While typically indicative of dementia, this approach inadvertently incorporates individuals with non-medication-treated dementia or off-label prescription of anti-dementia drugs for mild cognitive impairment. Thirdly, the single-center nature of our study curtailed our ability to track patients who were transferred to external healthcare facilities, potentially leading to an underestimation of dementia incidence post-transfer. Fourthly, this study used the onset of delirium as the cohort entry date and excluded patients who developed dementia within 180 days of the onset of delirium. Previous studies have also defined the time from the onset of delirium to the onset of cognitive decline and dementia as more than 3 months (28, 32) or more than 6 months (31, 33), but even 180 days may still be a short exclusion period. Fifthly, the influence of unmeasured confounding factors persists. Though we employed propensity score matching on a comprehensive array of over 90 covariates, the potential confounding impact of unmeasured variables remains beyond full mitigation (38). Sixthly, our study did not differentiate between subtypes of delirium (e.g., hyperactive, hypoactive, mixed). The inability to distinguish these subtypes may have influenced our findings, as different forms of delirium could contribute differently to the risk of dementia onset. Addressing these limitations would necessitate the execution of large-scale prospective trials.

## Conclusion

This study once again identifies a history of delirium as a risk factor for developing dementia. Whether delirium is a direct cause of dementia or merely a predictor of future dementia remains a matter of debate. If the former is the case, pharmacotherapy to prevent the onset of delirium from the beginning of hospitalization and adjustments to the hospital environment will greatly contribute to preventing the onset of dementia in the future. Even if the latter is the case, there are undoubtedly common physiological and pathological mechanisms between delirium and dementia, and many medical conditions and lifestyle habits that predispose to the development of delirium during hospitalization may, in the long run, lead to the development of new dementia, which, in turn, may be potential intervention targets to prevent the development of dementia. In any case, measures to prevent delirium during hospitalization have been discussed recently, but it is important to pay close attention to measures to prevent delirium from occurring not only during hospitalization, but also before hospitalization, and how to interact with patients. It has been said that patients with dementia have a high possibility of developing delirium, but the development of delirium has many negative effects on future medical care, such as an increased mortality rate, so the necessity and importance of measures to prevent delirium during hospitalization were reaffirmed.

## Data Availability

The original contributions presented in the study are included in the article/**Supplementary Material**. Further inquiries can be directed to the corresponding author.
