# Supplementary figures and images for: How much risk does delirium represent for the development of dementia?: Retrospective cohort study from over 260,000 patients record in a solitary institution
Source: Front Psychiatry. 2024 Sep 13;15:1387615. doi: 10.3389/fpsyt.2024.1387615 (PMC11427876; doi:10.3389/fpsyt.2024.1387615)

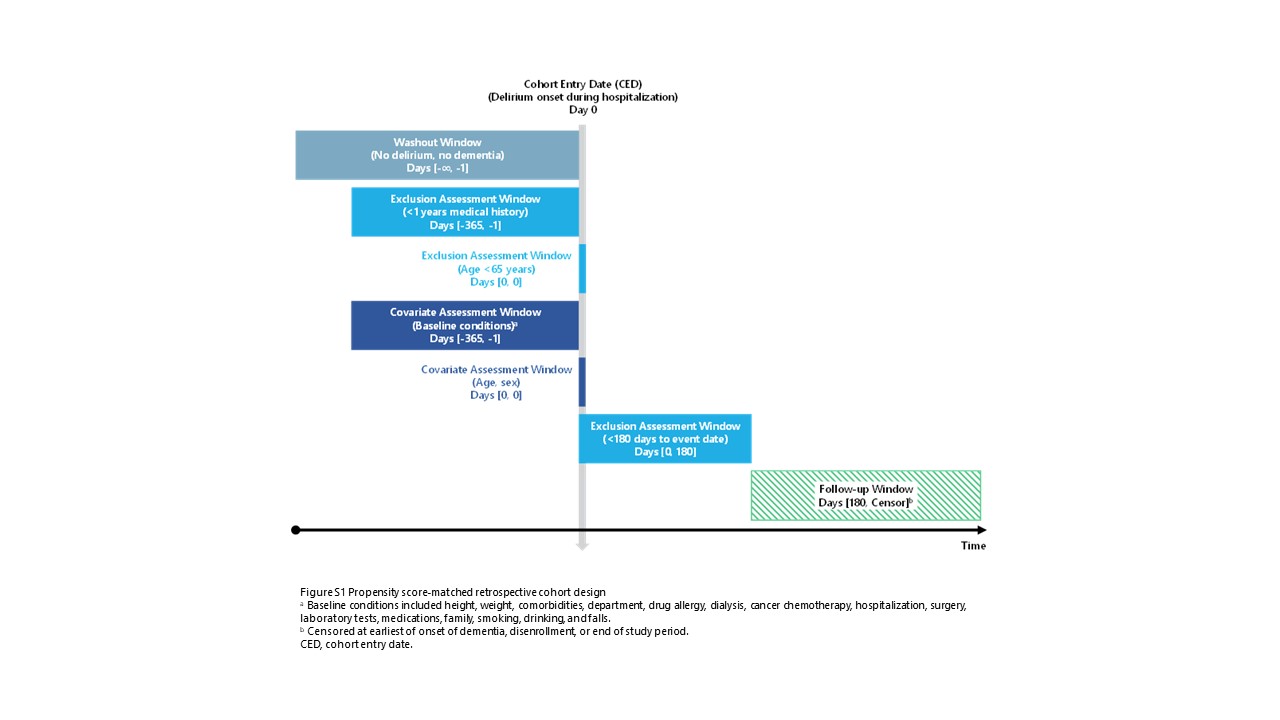

Supplement: Supplementary file 1 [file Image1.jpeg]
