# Supplementary material for: How much risk does delirium represent for the development of dementia?: Retrospective cohort study from over 260,000 patients record in a solitary institution
Source: Front Psychiatry. 2024 Sep 13;15:1387615. doi: 10.3389/fpsyt.2024.1387615 (PMC11427876; doi:10.3389/fpsyt.2024.1387615)
Supplement: Supplementary file 2 [file Table1.docx]

# Supplement

Table S1 List of ICD-10 codes for comorbidities

| Comorbidities | ICD-10 codes | Diagnoses |
| --- | --- | --- |
| Malignant neoplasms | C | Malignant neoplasms |
| Diabetes mellitus | E10 | Type 1 diabetes mellitus |
|  | E11 | Type 2 diabetes mellitus |
|  | E12 | Malnutrition-related diabetes mellitus |
|  | E13 | Other specified diabetes mellitus |
|  | E14 | Unspecified diabetes mellitus |
| Dyslipidemia | E78 | Disorders of lipoprotein metabolism and other lipidaemias |
| Schizophrenia | F20 | Schizophrenia |
| Depression | F32 | Depressive episode |
| Parkinson disease | G20 | Parkinson disease |
| Hypertension | I10 | Essential (primary) hypertension |
| Arrhythmias | I44 | Atrioventricular and left bundle-branch block |
|  | I45 | Other conduction disorders |
|  | I47 | Paroxysmal tachycardia |
|  | I48 | Atrial fibrillation and flutter |
|  | I49 | Other cardiac arrhythmias |
| Heart failure | I50 | Heart failure |
| COPD | J44 | Other chronic obstructive pulmonary disease |
| GERD | K21 | Gastro-oesophageal reflux disease |
| Constipation | K590 | Constipation |
| Rheumatoid arthritis | M05 | Seropositive rheumatoid arthritis |
|  | M06 | Other rheumatoid arthritis |
| Osteoporosis | M80 | Osteoporosis with pathological fracture |
|  | M81 | Osteoporosis without pathological fracture |
|  | M82 | Osteoporosis in diseases classified elsewhere |
| CKD | N18 | Chronic kidney disease |
| Overactive bladder | N31 | Neuromuscular dysfunction of bladder, not elsewhere classified |
| Prostatic hypertrophy | N40 | Hyperplasia of prostate |

Abbreviations: CKD, chronic kidney disease; COPD, chronic obstructive pulmonary disease; GERD, gastro esophageal reflux disease; ICD-10, International Statistical Classification of Diseases and Related Health Problems, 10th Revision.

Table S2 List of ATC codes for drugs

| Drugs | ATC codes | Drug class or names |
| --- | --- | --- |
| H_2_ receptor antagonist | A02BA | H_2_-receptor antagonists |
| Insulin | A10A | Insulins and analogues |
| Antidiabetic drug | A10B | Blood glucose lowering drugs, excl. insulins |
| Antithrombotic drug | B01 | Antithrombotic agents |
| Diuretic | C03CA | Sulfonamides, plain |
|  | C03DA | Aldosterone antagonists |
| Non-selective β-blocking drug | C07AA | Beta blocking agents, non-selective |
| Overactive bladder drug | G04BD | Drugs for urinary frequency and incontinence |
| Oral glucocorticoids | H02AB | Glucocorticoids |
| NSAIDs | M01A | Antiinflammatory and antirheumatic products, non-steroids |
|  | M02AA | Antiinflammatory preparations, non-steroids for topical use |
| Opioids | N02A | Opioids |
| Anticholinergic anti-Parkinson drug | N04AA | Tertiary amines |
| Antipsychotics | N05A | Antipsychotics |
| Benzodiazepine | N05B | Anxiolytics |
|  | N05C | Hypnotics and sedatives |
| Antidepressants | N06A | Antidepressants |
| Antihistamines | R06A | Antihistamines for systemic use |
| Antiemetic drug | A03FA01 | Metoclopramide |
|  | N05AB04 | Prochlorperazine |
|  | R06AD02 | Promethazine |

Abbreviations: ATC, Anatomical Therapeutic Chemical Classification System; NSAIDs, Non-steroidal antiinflammatory drugs.

Table S3 Characteristics of study patients

| Characteristics | Statistics | Overall | Delirium | Non-delirium | *p*-value | SMD |
| --- | --- | --- | --- | --- | --- | --- |
| Patients | n | 10781 | 582 | 10199 |  |  |
| Dementia | n (%) | 238 (2.2) | 32 (5.5) | 206 (2.0) | <0.001^*^ | 0.184 |
| Age, years | median (IQR) | 74 (70–79) | 78 (74–83) | 74 (70–78) | <0.001^*^ | 0.662 |
| Sex |  |  |  |  | 0.008^*^ | 0.115 |
| Female | n (%) | 4857 (45.1) | 231 (39.7) | 4626 (45.4) |  |  |
| Male | n (%) | 5924 (54.9) | 351 (60.3) | 5573 (54.6) |  |  |
| Height, cm | median (IQR) | 158.0 (151.0–164.8) | 157.0 (150.0–164.3) | 158.0 (151.0–164.8) | 0.027^*^ | 0.103 |
| Weight, kg | median (IQR) | 55.4 (47.8–63.3) | 51.5 (43.9–59.6) | 55.6 (48.0–63.5) | <0.001^*^ | 0.392 |
| BMI, kg/m^2^ | median (IQR) | 22.3 (19.8–24.8) | 20.8 (18.0–23.7) | 22.4 (19.9–24.8) | <0.001^*^ | 0.383 |
| Comorbidities |  |  |  |  |  |  |
| Arrhythmias | n (%) | 805 (7.5) | 90 (15.5) | 715 (7.0) | <0.001^*^ | 0.270 |
| CKD | n (%) | 290 (2.7) | 49 (8.4) | 241 (2.4) | <0.001^*^ | 0.271 |
| Constipation | n (%) | 802 (7.4) | 91 (15.6) | 711 (7.0) | <0.001^*^ | 0.276 |
| COPD | n (%) | 153 (1.4) | 22 (3.8) | 131 (1.3) | <0.001^*^ | 0.159 |
| Depression | n (%) | 120 (1.1) | 20 (3.4) | 100 (1.0) | <0.001^*^ | 0.168 |
| Diabetes mellitus | n (%) | 1594 (14.8) | 128 (22.0) | 1466 (14.4) | <0.001^*^ | 0.199 |
| Dyslipidemia | n (%) | 545 (5.1) | 50 (8.6) | 495 (4.9) | <0.001^*^ | 0.150 |
| GERD | n (%) | 752 (7.0) | 73 (12.5) | 679 (6.7) | <0.001^*^ | 0.201 |
| Heart failure | n (%) | 979 (9.1) | 117 (20.1) | 862 (8.5) | <0.001^*^ | 0.338 |
| Hypertension | n (%) | 1015 (9.4) | 104 (17.9) | 911 (8.9) | <0.001^*^ | 0.265 |
| Malignant neoplasms | n (%) | 2931 (27.2) | 208 (35.7) | 2723 (26.7) | <0.001^*^ | 0.196 |
| Osteoporosis | n (%) | 221 (2.0) | 30 (5.2) | 191 (1.9) | <0.001^*^ | 0.179 |
| Overactive bladder | n (%) | 119 (1.1) | 8 (1.4) | 111 (1.1) | 0.536 | 0.026 |
| Parkinson disease | n (%) | 76 (0.7) | 14 (2.4) | 62 (0.6) | <0.001^*^ | 0.148 |
| Prostatic hypertrophy | n (%) | 281 (2.6) | 16 (2.7) | 265 (2.6) | 0.789 | 0.009 |
| Rheumatoid arthritis | n (%) | 188 (1.7) | 21 (3.6) | 167 (1.6) | 0.002^*^ | 0.124 |
| Schizophrenia | n (%) | 68 (0.6) | 25 (4.3) | 43 (0.4) | <0.001^*^ | 0.257 |
| Number of department | median (IQR) | 2 (2–4) | 4 (3–6) | 2 (2–4) | <0.001^*^ | 0.958 |
| Department |  |  |  |  |  |  |
| Anesthesiology | n (%) | 683 (6.3) | 55 (9.5) | 628 (6.2) | 0.003^*^ | 0.123 |
| Breast surgery | n (%) | 479 (4.4) | 17 (2.9) | 462 (4.5) | 0.078 | 0.085 |
| Cardiology | n (%) | 3409 (31.6) | 277 (47.6) | 3132 (30.7) | <0.001^*^ | 0.351 |
| Cardiovascular surgery | n (%) | 871 (8.1) | 94 (16.2) | 777 (7.6) | <0.001^*^ | 0.266 |
| Dentistry | n (%) | 2040 (18.9) | 229 (39.3) | 1811 (17.8) | <0.001^*^ | 0.492 |
| Dermatology | n (%) | 1204 (11.2) | 114 (19.6) | 1090 (10.7) | <0.001^*^ | 0.250 |
| Diabetology | n (%) | 1271 (11.8) | 102 (17.5) | 1169 (11.5) | <0.001^*^ | 0.173 |
| Emergency | n (%) | 342 (3.2) | 116 (19.9) | 226 (2.2) | <0.001^*^ | 0.588 |
| Gastroenterology | n (%) | 3064 (28.4) | 217 (37.3) | 2847 (27.9) | <0.001^*^ | 0.201 |
| Gastrointestinal surgery | n (%) | 1618 (15.0) | 132 (22.7) | 1486 (14.6) | <0.001^*^ | 0.209 |
| General medicine | n (%) | 451 (4.2) | 74 (12.7) | 377 (3.7) | <0.001^*^ | 0.333 |
| Gynecology | n (%) | 575 (5.3) | 19 (3.3) | 556 (5.5) | 0.022^*^ | 0.107 |
| Hematology | n (%) | 511 (4.7) | 33 (5.7) | 478 (4.7) | 0.270 | 0.044 |
| Nephrology | n (%) | 340 (3.2) | 31 (5.3) | 309 (3.0) | 0.005^*^ | 0.115 |
| Neurology | n (%) | 553 (5.1) | 67 (11.5) | 486 (4.8) | <0.001^*^ | 0.249 |
| Neurosurgery | n (%) | 967 (9.0) | 102 (17.5) | 865 (8.5) | <0.001^*^ | 0.271 |
| Obstetrics | n (%) | 25 (0.2) | 1 (0.2) | 24 (0.2) | 1.000 | 0.014 |
| Ophthalmology | n (%) | 1603 (14.9) | 109 (18.7) | 1494 (14.6) | 0.008^*^ | 0.110 |
| Orthopedic surgery | n (%) | 1812 (16.8) | 132 (22.7) | 1680 (16.5) | <0.001^*^ | 0.157 |
| Otorhinolaryngology | n (%) | 1360 (12.6) | 112 (19.2) | 1248 (12.2) | <0.001^*^ | 0.193 |
| Pediatrics | n (%) | 1 (0.0) | 0 (0.0) | 1 (0.0) | 1.000 | 0.014 |
| Plastic surgery | n (%) | 416 (3.9) | 29 (5.0) | 387 (3.8) | 0.150 | 0.058 |
| Psychiatry | n (%) | 358 (3.3) | 72 (12.4) | 286 (2.8) | <0.001^*^ | 0.367 |
| Radiology | n (%) | 611 (5.7) | 33 (5.7) | 578 (5.7) | 1.000 | <0.001 |
| Rehabilitation | n (%) | 922 (8.6) | 266 (45.7) | 656 (6.4) | <0.001^*^ | 1.000 |
| Respiratory | n (%) | 1400 (13.0) | 95 (16.3) | 1305 (12.8) | 0.016^*^ | 0.100 |
| Respiratory surgery | n (%) | 603 (5.6) | 42 (7.2) | 561 (5.5) | 0.094 | 0.070 |
| Rheumatology | n (%) | 878 (8.1) | 59 (10.1) | 819 (8.0) | 0.073 | 0.073 |
| Urology | n (%) | 2409 (22.3) | 155 (26.6) | 2254 (22.1) | 0.012^*^ | 0.106 |
| Dialysis | n (%) | 151 (1.4) | 47 (8.1) | 104 (1.0) | <0.001^*^ | 0.344 |
| Chemotherapy | n (%) | 698 (6.5) | 41 (7.0) | 657 (6.4) | 0.545 | 0.024 |
| Number of hospitalization | median (IQR) | 0 (0–0) | 1 (1–2) | 0 (0–0) | <0.001^*^ | 1.102 |
| Number of surgeries | median (IQR) | 0 (0–0) | 0 (0–1) | 0 (0–0) | <0.001^*^ | 0.461 |
| Clinical laboratory test |  |  |  |  |  |  |
| Albumin, g/dL | median (IQR) | 4.0 (3.7–4.2) | 3.1 (2.6–3.6) | 4.0 (3.8–4.3) | <0.001^*^ | 1.608 |
| ALT, U/L | median (IQR) | 17 (12–24) | 17 (11–32) | 17 (13–23) | 0.113 | 0.157 |
| AST, U/L | median (IQR) | 22 (18–28) | 25 (19–37) | 22 (18–28) | <0.001^*^ | 0.171 |
| BUN, mg/dL | median (IQR) | 17 (14–21) | 19 (13–27) | 17 (14–21) | <0.001^*^ | 0.363 |
| Creatinine, mg/dL | median (IQR) | 0.8 (0.7–1.0) | 0.9 (0.7–1.3) | 0.8 (0.7–1.0) | <0.001^*^ | 0.317 |
| Hemoglobin, g/dL | median (IQR) | 12.9 (11.8–14.0) | 11.1 (9.7–12.5) | 13.0 (11.9–14.0) | <0.001^*^ | 0.953 |
| Leukocyte, 10^3^/μL | median (IQR) | 5.7 (4.6–7.0) | 7.0 (5.3–9.5) | 5.6 (4.6–6.9) | <0.001^*^ | 0.618 |
| Neutrophil, /μL | median (IQR) | 3428 (2629–4505) | 5201 (3576–7658) | 3384 (2607–4398) | <0.001^*^ | 0.831 |
| Platelet, 10^3^/μL | median (IQR) | 207 (168–251) | 185 (137–240) | 208 (170–251) | <0.001^*^ | 0.188 |
| Total bilirubin, mg/dL | median (IQR) | 0.5 (0.4–0.7) | 0.6 (0.4–0.9) | 0.5 (0.4–0.7) | <0.001^*^ | 0.229 |
| Total lymphocyte, /μL | median (IQR) | 1440 (1072–1854) | 932 (638–1334) | 1466 (1103–1873) | <0.001^*^ | 0.681 |
| Packaging | n (%) | 5259 (48.8) | 547 (94.0) | 4712 (46.2) | <0.001^*^ | 1.224 |
| Number of bring drug | median (IQR) | 0 (0–0) | 0 (0–10) | 0 (0–0) | <0.001^*^ | 0.837 |
| Number of drug | median (IQR) | 5 (1–12) | 16 (9–24) | 4 (0–11) | <0.001^*^ | 1.047 |
| Drugs |  |  |  |  |  |  |
| H_2_ receptor antagonist | n (%) | 776 (7.2) | 85 (14.6) | 691 (6.8) | <0.001^*^ | 0.255 |
| Insulin | n (%) | 620 (5.8) | 133 (22.9) | 487 (4.8) | <0.001^*^ | 0.543 |
| Antidiabetic drug | n (%) | 1233 (11.4) | 125 (21.5) | 1108 (10.9) | <0.001^*^ | 0.291 |
| Antithrombotic drug | n (%) | 3350 (31.1) | 350 (60.1) | 3000 (29.4) | <0.001^*^ | 0.650 |
| Diuretic | n (%) | 987 (9.2) | 155 (26.6) | 832 (8.2) | <0.001^*^ | 0.503 |
| Non-selective β-blocking drug | n (%) | 12 (0.1) | 2 (0.3) | 10 (0.1) | 0.134 | 0.052 |
| Overactive bladder drug | n (%) | 347 (3.2) | 42 (7.2) | 305 (3.0) | <0.001^*^ | 0.193 |
| Oral glucocorticoids | n (%) | 1104 (10.2) | 101 (17.4) | 1003 (9.8) | <0.001^*^ | 0.221 |
| NSAIDs | n (%) | 3325 (30.8) | 326 (56.0) | 2999 (29.4) | <0.001^*^ | 0.559 |
| Opioids | n (%) | 65 (0.6) | 10 (1.7) | 55 (0.5) | 0.002^*^ | 0.112 |
| Anticholinergic anti-Parkinson drug | n (%) | 23 (0.2) | 7 (1.2) | 16 (0.2) | <0.001^*^ | 0.128 |
| Antipsychotics | n (%) | 467 (4.3) | 164 (28.2) | 303 (3.0) | <0.001^*^ | 0.741 |
| Benzodiazepine | n (%) | 1606 (14.9) | 243 (41.8) | 1363 (13.4) | <0.001^*^ | 0.670 |
| Antidepressants | n (%) | 494 (4.6) | 101 (17.4) | 393 (3.9) | <0.001^*^ | 0.449 |
| Antihistamines | n (%) | 102 (0.9) | 11 (1.9) | 91 (0.9) | 0.025^*^ | 0.085 |
| Antiemetic drug | n (%) | 431 (4.0) | 47 (8.1) | 384 (3.8) | <0.001^*^ | 0.183 |
| Diazepam equivalence, mg | median (IQR) | 0.0 (0.0–0.0) | 0.0 (0.0–5.3) | 0.0 (0.0–0.0) | <0.001^*^ | 0.559 |
| Chlorpromazine equivalence, mg | median (IQR) | 0.0 (0.0–0.0) | 0.0 (0.0–37.9) | 0.0 (0.0–0.0) | <0.001^*^ | 0.484 |
| Number of family | median (IQR) | 3 (2–3) | 3 (2–3) | 3 (2–3) | 0.772 | 0.002 |
| Family |  |  |  |  |  |  |
| Parent | n (%) | 358 (3.3) | 16 (2.7) | 342 (3.4) | 0.551 | 0.035 |
| Sibling | n (%) | 880 (8.2) | 45 (7.7) | 835 (8.2) | 0.756 | 0.017 |
| Son | n (%) | 9561 (88.7) | 529 (90.9) | 9032 (88.6) | 0.092 | 0.077 |
| Spouse | n (%) | 9794 (90.8) | 522 (89.7) | 9272 (90.9) | 0.336 | 0.041 |
| History of smoking | n (%) | 4913 (45.6) | 294 (50.5) | 4619 (45.3) | 0.015^*^ | 0.105 |
| Drinking | n (%) | 3346 (31.0) | 151 (25.9) | 3195 (31.3) | 0.007^*^ | 0.119 |
| Fall | n (%) | 80 (0.7) | 31 (5.3) | 49 (0.5) | <0.001^*^ | 0.292 |

Abbreviations: ALT, alanine aminotransferase; AST, aspartate aminotransferase; BMI, body mass index; BUN, blood urea nitrogen; CKD, chronic kidney disease; COPD, chronic obstructive pulmonary disease; GERD, gastroesophageal reflux disease; IQR, interquartile range; NSAIDs, non-steroidal anti-inflammatory drug; SMD, standardized mean difference. ^*^*p* < 0.05.

Table S4 Characteristics of study patients after propensity score matching

| Characteristics | Statistics | Delirium | Non-delirium | *p*-value | SMD |
| --- | --- | --- | --- | --- | --- |
| Patients | n | 288 | 288 |  |  |
| Dementia | n (%) | 25 (8.7) | 7 (2.4) | 0.002^*^ | 0.275 |
| Age, years | median (IQR) | 78 (73–83) | 78 (73–82) | 0.597 | 0.058 |
| Sex |  |  |  | 0.932 | 0.014 |
| Female | n (%) | 119 (41.3) | 117 (40.6) |  |  |
| Male | n (%) | 169 (58.7) | 171 (59.4) |  |  |
| Height, cm | median (IQR) | 157.0 (150.0–164.5) | 158.0 (150.6–163.5) | 0.797 | 0.018 |
| Weight, kg | median (IQR) | 52.8 (44.9–60.3) | 52.5 (45.0–59.0) | 0.953 | 0.007 |
| BMI, kg/m^2^ | median (IQR) | 21.4 (18.8–24.1) | 21.0 (18.8–23.6) | 0.712 | 0.002 |
| Comorbidities |  |  |  |  |  |
| Arrhythmias | n (%) | 35 (12.2) | 34 (11.8) | 1.000 | 0.011 |
| CKD | n (%) | 19 (6.6) | 15 (5.2) | 0.596 | 0.059 |
| Constipation | n (%) | 51 (17.7) | 39 (13.5) | 0.207 | 0.115 |
| COPD | n (%) | 12 (4.2) | 13 (4.5) | 1.000 | 0.017 |
| Depression | n (%) | 10 (3.5) | 7 (2.4) | 0.624 | 0.062 |
| Diabetes mellitus | n (%) | 50 (17.4) | 40 (13.9) | 0.302 | 0.096 |
| Dyslipidemia | n (%) | 22 (7.6) | 18 (6.2) | 0.623 | 0.055 |
| GERD | n (%) | 40 (14.6) | 36 (12.5) | 0.712 | 0.041 |
| Heart failure | n (%) | 42 (14.6) | 40 (13.9) | 0.905 | 0.020 |
| Hypertension | n (%) | 41 (14.2) | 38 (13.2) | 0.809 | 0.030 |
| Malignant neoplasms | n (%) | 98 (34.0) | 110 (38.2) | 0.340 | 0.087 |
| Osteoporosis | n (%) | 17 (5.9) | 12 (4.2) | 0.446 | 0.079 |
| Overactive bladder | n (%) | 5 (1.7) | 1 (0.3) | 0.216 | 0.137 |
| Parkinson disease | n (%) | 5 (1.7) | 9 (3.1) | 0.418 | 0.090 |
| Prostatic hypertrophy | n (%) | 8 (2.8) | 5 (1.7) | 0.577 | 0.070 |
| Rheumatoid arthritis | n (%) | 13 (4.5) | 11 (3.8) | 0.835 | 0.035 |
| Schizophrenia | n (%) | 10 (3.5) | 9 (3.1) | 1.000 | 0.019 |
| Number of department | median (IQR) | 4 (2–5) | 3 (2–5) | 0.280 | 0.106 |
| Department |  |  |  |  |  |
| Anesthesiology | n (%) | 25 (8.7) | 24 (8.3) | 1.000 | 0.012 |
| Breast surgery | n (%) | 9 (3.1) | 9 (3.1) | 1.000 | <0.001 |
| Cardiology | n (%) | 121 (42.0) | 110 (38.2) | 0.395 | 0.078 |
| Cardiovascular surgery | n (%) | 38 (13.2) | 31 (10.8) | 0.442 | 0.075 |
| Dentistry | n (%) | 84 (29.2) | 83 (28.8) | 1.000 | 0.008 |
| Dermatology | n (%) | 55 (19.1) | 41 (14.2) | 0.146 | 0.131 |
| Diabetology | n (%) | 44 (15.3) | 38 (13.2) | 0.551 | 0.060 |
| Emergency | n (%) | 36 (12.5) | 36 (12.5) | 1.000 | <0.001 |
| Gastroenterology | n (%) | 101 (35.1) | 100 (34.7) | 1.000 | 0.007 |
| Gastrointestinal surgery | n (%) | 47 (16.3) | 59 (20.5) | 0.237 | 0.108 |
| General medicine | n (%) | 32 (11.1) | 28 (9.7) | 0.683 | 0.045 |
| Gynecology | n (%) | 11 (3.8) | 13 (4.5) | 0.835 | 0.035 |
| Hematology | n (%) | 20 (6.9) | 22 (7.6) | 0.873 | 0.027 |
| Nephrology | n (%) | 16 (5.6) | 12 (4.2) | 0.562 | 0.065 |
| Neurology | n (%) | 22 (7.6) | 29 (10.1) | 0.379 | 0.086 |
| Neurosurgery | n (%) | 42 (14.6) | 39 (13.5) | 0.811 | 0.030 |
| Obstetrics | n (%) | 1 (0.3) | 0 (0.0) | 1.000 | 0.083 |
| Ophthalmology | n (%) | 56 (19.4) | 47 (16.3) | 0.384 | 0.082 |
| Orthopedic surgery | n (%) | 65 (22.6) | 53 (18.4) | 0.256 | 0.103 |
| Otorhinolaryngology | n (%) | 55 (19.1) | 63 (21.9) | 0.470 | 0.069 |
| Pediatrics | n (%) | 0 (0.0) | 0 (0.0) | N.D. | <0.001 |
| Plastic surgery | n (%) | 17 (5.9) | 14 (4.9) | 0.713 | 0.046 |
| Psychiatry | n (%) | 31 (10.8) | 30 (10.4) | 1.000 | 0.011 |
| Radiology | n (%) | 14 (4.9) | 11 (3.8) | 0.683 | 0.051 |
| Rehabilitation | n (%) | 84 (29.2) | 70 (24.3) | 0.221 | 0.110 |
| Respiratory | n (%) | 43 (14.9) | 40 (13.9) | 0.813 | 0.030 |
| Respiratory surgery | n (%) | 20 (6.9) | 21 (7.3) | 1.000 | 0.014 |
| Rheumatology | n (%) | 37 (12.8) | 33 (11.5) | 0.702 | 0.043 |
| Urology | n (%) | 71 (24.7) | 66 (22.9) | 0.696 | 0.041 |
| Dialysis | n (%) | 11 (3.8) | 5 (1.7) | 0.204 | 0.127 |
| Chemotherapy | n (%) | 30 (10.4) | 30 (10.4) | 1.000 | <0.001 |
| Number of hospitalization | median (IQR) | 1 (1–2) | 0 (0–2) | <0.001^*^ | 0.025 |
| Number of surgeries | median (IQR) | 0 (0–1) | 0 (0–0) | 0.190 | 0.071 |
| Clinical laboratory test |  |  |  |  |  |
| Albumin, g/dL | median (IQR) | 3.5 (2.9–3.9) | 3.5 (3.0–3.9) | 0.600 | 0.035 |
| ALT, U/L | median (IQR) | 16 (11–27) | 17 (12–27) | 0.354 | 0.056 |
| AST, U/L | median (IQR) | 22 (18–31) | 24 (18–34) | 0.252 | 0.063 |
| BUN, mg/dL | median (IQR) | 19 (13–27) | 18 (14–24) | 0.722 | 0.137 |
| Creatinine, mg/dL | median (IQR) | 0.9 (0.7–1.3) | 0.9 (0.7–1.2) | 0.495 | 0.169 |
| Hemoglobin, g/dL | median (IQR) | 11.5 (10.1–12.8) | 11.8 (10.3–12.8) | 0.400 | 0.058 |
| Leukocyte, 10^3^/μL | median (IQR) | 6.1 (4.9–7.8) | 5.8 (4.3–7.8) | 0.134 | 0.021 |
| Neutrophil, /μL | median (IQR) | 4203 (3061–5875) | 3880 (2565–5693) | 0.082 | 0.033 |
| Platelet, 10^3^/μL | median (IQR) | 196 (152–248) | 191 (149–254) | 0.718 | 0.034 |
| Total bilirubin, mg/dL | median (IQR) | 0.6 (0.4–0.8) | 0.5 (0.4–0.8) | 0.126 | 0.073 |
| Total lymphocyte, /μL | median (IQR) | 1073 (771–1501) | 1120 (804–1516) | 0.707 | 0.013 |
| Packaging | n (%) | 261 (90.6) | 258 (89.6) | 0.780 | 0.035 |
| Number of bring drug | median (IQR) | 0 (0–7) | 0 (0–3) | 0.215 | 0.002 |
| Number of drug | median (IQR) | 13 (7–23) | 11 (4–21) | 0.021^*^ | 0.146 |
| Drugs |  |  |  |  |  |
| H_2_ receptor antagonist | n (%) | 41 (14.2) | 31 (10.8) | 0.257 | 0.105 |
| Insulin | n (%) | 42 (14.6) | 40 (13.9) | 0.905 | 0.020 |
| Antidiabetic drug | n (%) | 48 (16.7) | 45 (15.6) | 0.821 | 0.028 |
| Antithrombotic drug | n (%) | 152 (52.8) | 137 (47.6) | 0.243 | 0.104 |
| Diuretic | n (%) | 64 (22.2) | 63 (21.9) | 1.000 | 0.008 |
| Non-selective β-blocking drug | n (%) | 1 (0.3) | 2 (0.7) | 1.000 | 0.048 |
| Overactive bladder drug | n (%) | 15 (5.2) | 17 (5.9) | 0.856 | 0.030 |
| Oral glucocorticoids | n (%) | 58 (20.1) | 44 (15.3) | 0.156 | 0.128 |
| NSAIDs | n (%) | 153 (53.1) | 157 (54.5) | 0.802 | 0.028 |
| Opioids | n (%) | 7 (2.4) | 5 (1.7) | 0.772 | 0.049 |
| Anticholinergic anti-Parkinson drug | n (%) | 3 (1.0) | 5 (1.7) | 0.725 | 0.059 |
| Antipsychotics | n (%) | 65 (22.6) | 64 (22.2) | 1.000 | 0.008 |
| Benzodiazepine | n (%) | 106 (36.8) | 103 (35.8) | 0.862 | 0.022 |
| Antidepressants | n (%) | 45 (15.6) | 50 (17.4) | 0.654 | 0.047 |
| Antihistamines | n (%) | 7 (2.4) | 3 (1.0) | 0.339 | 0.106 |
| Antiemetic drug | n (%) | 28 (9.7) | 29 (10.1) | 1.000 | 0.012 |
| Diazepam equivalence , mg | median (IQR) | 0.0 (0.0–5.0) | 0.0 (0.0–4.8) | 0.281 | 0.024 |
| Chlorpromazine equivalence, mg | median (IQR) | 0.0 (0.0–0.0) | 0.0 (0.0–0.0) | 0.754 | 0.044 |
| Number of family | median (IQR) | 3 (2–3) | 3 (2–3) | 0.988 | 0.035 |
| Family |  |  |  |  |  |
| Parent | n (%) | 7 (2.4) | 5 (1.7) | 0.772 | 0.049 |
| Sibling | n (%) | 20 (6.9) | 18 (6.2) | 0.867 | 0.028 |
| Son | n (%) | 259 (89.9) | 263 (91.3) | 0.668 | 0.048 |
| Spouse | n (%) | 257 (89.2) | 256 (88.9) | 1.000 | 0.011 |
| History of smoking | n (%) | 135 (46.9) | 136 (47.2) | 1.000 | 0.007 |
| Drinking | n (%) | 74 (25.7) | 77 (26.7) | 0.850 | 0.024 |
| Fall | n (%) | 28 (9.7) | 9 (3.1) | 0.002^*^ | 0.272 |

Abbreviations: ALT, alanine aminotransferase; AST, aspartate aminotransferase; BMI, body mass index; BUN, blood urea nitrogen; CKD, chronic kidney disease; COPD, chronic obstructive pulmonary disease; GERD, gastroesophageal reflux disease; IQR, interquartile range; N.D., not determined; NSAIDs, non-steroidal anti-inflammatory drug; SMD, standardized mean difference. ^*^*p* < 0.05.

Table S5 Cox regression analysis to estimate the hazard ratios for developing dementia

|  | Unadjusted | | |  | Adjusted | | | |
| --- | --- | --- | --- | --- | --- | --- | --- | --- |
| Variables | HR | 95% CI | *p*-value |  | HR | 95% CI | *p*-value | VIF |
| Delirium | 4.96 | 3.40–7.23 | <0.001^*^ |  | 2.90 | 1.80–4.68 | <0.001^*^ | 1.60 |
| Age, years (per 1 unit) | 1.10 | 1.08–1.12 | <0.001^*^ |  | 1.08 | 1.06–1.10 | <0.001^*^ | 1.11 |
| Sex (male) | 0.85 | 0.66–1.09 | 0.195 |  |  |  |  |  |
| Height, cm (per 1 unit) | 0.98 | 0.96–0.99 | 0.001^*^ |  |  |  |  |  |
| Weight, kg (per 1 unit) | 0.96 | 0.95–0.97 | <0.001^*^ |  |  |  |  |  |
| BMI, kg/m^2^ (per 1 unit) | 0.91 | 0.88–0.94 | <0.001^*^ |  | 0.93 | 0.90–0.97 | <0.001^*^ | 1.08 |
| Comorbidities |  |  |  |  |  |  |  |  |
| Arrhythmias | 0.95 | 0.57–1.57 | 0.834 |  |  |  |  |  |
| CKD | 1.61 | 0.80–3.27 | 0.183 |  |  |  |  |  |
| Constipation | 1.22 | 0.75–1.97 | 0.416 |  |  |  |  |  |
| COPD | 1.42 | 0.53–3.80 | 0.491 |  |  |  |  |  |
| Depression | 4.69 | 2.56–8.60 | <0.001^*^ |  | 3.45 | 1.64–7.26 | 0.001^*^ | 1.51 |
| Diabetes mellitus | 1.44 | 1.04–1.99 | 0.029^*^ |  | 1.26 | 0.89–1.79 | 0.200 | 1.17 |
| Dyslipidemia | 1.06 | 0.59–1.89 | 0.854 |  |  |  |  |  |
| GERD | 1.19 | 0.74–1.93 | 0.477 |  |  |  |  |  |
| Heart failure | 1.68 | 1.16–2.44 | 0.006^*^ |  | 1.33 | 0.89–1.99 | 0.162 | 1.16 |
| Hypertension | 1.45 | 0.99–2.14 | 0.059 |  |  |  |  |  |
| Malignant neoplasms | 0.65 | 0.46–0.92 | 0.015^*^ |  | 0.77 | 0.53–1.12 | 0.171 | 1.18 |
| Osteoporosis | 1.02 | 0.42–2.46 | 0.973 |  |  |  |  |  |
| Overactive bladder | 0.73 | 0.18–2.94 | 0.660 |  |  |  |  |  |
| Parkinson disease | N.D. | N.D. | N.D. |  |  |  |  |  |
| Prostatic hypertrophy | 0.33 | 0.08–1.31 | 0.114 |  |  |  |  |  |
| Rheumatoid arthritis | 1.01 | 0.42–2.46 | 0.979 |  |  |  |  |  |
| Schizophrenia | 2.32 | 0.74–7.25 | 0.147 |  |  |  |  |  |
| Number of department (per 1 unit) | 1.15 | 1.08–1.23 | <0.001^*^ |  | 0.98 | 0.88–1.08 | 0.622 | 2.51 |
| Department |  |  |  |  |  |  |  |  |
| Anesthesiology | 0.67 | 0.36–1.27 | 0.223 |  |  |  |  |  |
| Breast surgery | 1.25 | 0.74–2.11 | 0.399 |  |  |  |  |  |
| Cardiology | 1.04 | 0.80–1.36 | 0.753 |  |  |  |  |  |
| Cardiovascular surgery | 1.23 | 0.82–1.87 | 0.320 |  |  |  |  |  |
| Dentistry | 1.19 | 0.85–1.67 | 0.309 |  |  |  |  |  |
| Dermatology | 1.74 | 1.26–2.40 | 0.001^*^ |  | 1.51 | 1.03–2.20 | 0.033^*^ | 1.38 |
| Diabetology | 1.50 | 1.08–2.09 | 0.016^*^ |  | 1.32 | 0.89–1.97 | 0.168 | 1.44 |
| Emergency | 1.71 | 0.91–3.22 | 0.097 |  |  |  |  |  |
| Gastroenterology | 0.95 | 0.71–1.26 | 0.716 |  |  |  |  |  |
| Gastrointestinal surgery | 0.56 | 0.36–0.89 | 0.014^*^ |  | 0.72 | 0.44–1.18 | 0.192 | 1.15 |
| General medicine | 1.64 | 0.98–2.77 | 0.062 |  |  |  |  |  |
| Gynecology | 0.94 | 0.53–1.69 | 0.846 |  |  |  |  |  |
| Hematology | 1.06 | 0.56–2.00 | 0.855 |  |  |  |  |  |
| Nephrology | 1.52 | 0.81–2.87 | 0.192 |  |  |  |  |  |
| Neurology | 1.18 | 0.70–1.99 | 0.533 |  |  |  |  |  |
| Neurosurgery | 2.18 | 1.57–3.03 | <0.001^*^ |  |  |  |  |  |
| Obstetrics | 1.26 | 0.18–9.00 | 0.816 |  |  |  |  |  |
| Ophthalmology | 1.39 | 1.02–1.87 | 0.034^*^ |  | 1.15 | 0.83–1.60 | 0.405 | 1.21 |
| Orthopedic surgery | 1.03 | 0.74–1.44 | 0.839 |  |  |  |  |  |
| Otorhinolaryngology | 0.97 | 0.66–1.41 | 0.870 |  |  |  |  |  |
| Pediatrics | N.D. | N.D. | N.D. |  |  |  |  |  |
| Plastic surgery | 0.94 | 0.49–1.84 | 0.866 |  |  |  |  |  |
| Psychiatry | 3.30 | 2.20–4.96 | <0.001^*^ |  | 2.91 | 1.75–4.86 | <0.001^*^ | 1.58 |
| Radiology | 0.78 | 0.39–1.58 | 0.497 |  |  |  |  |  |
| Rehabilitation | 1.86 | 1.27–2.73 | 0.001^*^ |  |  |  |  |  |
| Respiratory | 1.63 | 1.17–2.27 | 0.004^*^ |  | 1.45 | 1.01–2.08 | 0.044^*^ | 1.19 |
| Respiratory surgery | 0.70 | 0.36–1.36 | 0.295 |  |  |  |  |  |
| Rheumatology | 1.37 | 0.93–2.01 | 0.116 |  |  |  |  |  |
| Urology | 0.78 | 0.56–1.08 | 0.139 |  |  |  |  |  |
| Dialysis | 1.67 | 0.62–4.48 | 0.312 |  |  |  |  |  |
| Chemotherapy | 0.61 | 0.27–1.37 | 0.233 |  |  |  |  |  |
| Number of hospitalization (per 1 unit) | 1.04 | 0.93–1.18 | 0.477 |  |  |  |  |  |
| Number of surgeries (per 1 unit) | 0.65 | 0.43–0.99 | 0.042^*^ |  | 0.48 | 0.30–0.77 | 0.002^*^ | 1.22 |
| Clinical laboratory test |  |  |  |  |  |  |  |  |
| Albumin, g/dL (per 1 unit) | 0.66 | 0.51–0.85 | 0.001^*^ |  |  |  |  |  |
| ALT, U/L (per 1 unit) | 1.00 | 1.00–1.00 | 0.588 |  |  |  |  |  |
| AST, U/L (per 1 unit) | 1.00 | 1.00–1.00 | 0.062 |  |  |  |  |  |
| BUN, mg/dL (per 1 unit) | 1.01 | 1.00–1.02 | 0.096 |  |  |  |  |  |
| Creatinine, mg/dL (per 1 unit) | 1.08 | 0.98–1.19 | 0.113 |  |  |  |  |  |
| Hemoglobin, g/dL (per 1 unit) | 0.86 | 0.80–0.92 | <0.001^*^ |  | 0.97 | 0.89–1.05 | 0.449 | 1.28 |
| Leukocyte, 10^3^/μL (per 1 unit) | 1.06 | 1.03–1.10 | <0.001^*^ |  |  |  |  |  |
| Neutrophil, /μL (per 1 unit) | 1.00 | 1.00–1.00 | <0.001^*^ |  | 1.00 | 1.00–1.00 | 0.058 | 1.16 |
| Platelet, 10^3^/μL (per 1 unit) | 1.00 | 1.00–1.00 | 0.085 |  |  |  |  |  |
| Total bilirubin, mg/dL (per 1 unit) | 0.91 | 0.63–1.33 | 0.632 |  |  |  |  |  |
| Total lymphocyte, /μL (per 1 unit) | 1.00 | 1.00–1.00 | 0.003^*^ |  | 1.00 | 1.00–1.00 | 0.540 | 1.15 |
| Packaging | 6.85 | 4.81–9.77 | <0.001^*^ |  |  |  |  |  |
| Numberof bring drug (per 1 unit) | 1.02 | 0.96–1.08 | 0.520 |  |  |  |  |  |
| Number of drug (per 1 unit) | 1.03 | 1.02–1.04 | <0.001^*^ |  | 1.00 | 0.98–1.02 | 0.873 | 3.03 |
| Drugs |  |  |  |  |  |  |  |  |
| H_2_ receptor antagonist | 0.98 | 0.62–1.55 | 0.931 |  |  |  |  |  |
| Insulin | 1.46 | 0.92–2.34 | 0.112 |  |  |  |  |  |
| Antidiabetic drug | 1.47 | 1.04–2.07 | 0.028^*^ |  | 1.10 | 0.73–1.66 | 0.657 | 1.46 |
| Antithrombotic drug | 1.38 | 1.06–1.78 | 0.016^*^ |  | 0.92 | 0.67–1.26 | 0.598 | 1.44 |
| Diuretic | 1.75 | 1.21–2.53 | 0.003^*^ |  | 1.15 | 0.76–1.74 | 0.517 | 1.28 |
| Non-selective β-blocking drug | N.D. | N.D. | N.D. |  |  |  |  |  |
| Overactive bladder drug | 1.80 | 1.07–3.04 | 0.027^*^ |  | 1.41 | 0.82–2.43 | 0.216 | 1.08 |
| Oral glucocorticoids | 1.03 | 0.67–1.57 | 0.901 |  |  |  |  |  |
| NSAIDs | 1.11 | 0.84–1.46 | 0.457 |  |  |  |  |  |
| Opioids | N.D. | N.D. | N.D. |  |  |  |  |  |
| Anticholinergic anti-Parkinson drug | N.D. | N.D. | N.D. |  |  |  |  |  |
| Antipsychotics | 2.13 | 1.30–3.49 | 0.003^*^ |  | 0.68 | 0.37–1.24 | 0.207 | 1.49 |
| Benzodiazepine | 1.31 | 0.94–1.81 | 0.110 |  |  |  |  |  |
| Antidepressants | 2.07 | 1.33–3.25 | 0.001^*^ |  | 0.69 | 0.37–1.27 | 0.232 | 1.85 |
| Antihistamines | 1.36 | 0.43–4.24 | 0.600 |  |  |  |  |  |
| Antiemetic drug | 0.85 | 0.38–1.90 | 0.684 |  |  |  |  |  |
| Diazepam equivalence, mg (per 1 unit) | 1.00 | 0.97–1.03 | 0.948 |  |  |  |  |  |
| Chlorpromazine equivalence, mg (per 1 unit) | 1.00 | 1.00–1.00 | 0.266 |  |  |  |  |  |
| Number of family (per 1 unit) | 1.08 | 0.96–1.21 | 0.187 |  |  |  |  |  |
| Family |  |  |  |  |  |  |  |  |
| Parent | 1.30 | 0.67–2.54 | 0.436 |  |  |  |  |  |
| Sibling | 0.78 | 0.46–1.32 | 0.359 |  |  |  |  |  |
| Son | 1.58 | 0.98–2.55 | 0.063 |  |  |  |  |  |
| Spouse | 1.30 | 0.79–2.13 | 0.296 |  |  |  |  |  |
| History of smoking | 0.83 | 0.64–1.08 | 0.167 |  |  |  |  |  |
| Drinking | 0.88 | 0.67–1.17 | 0.380 |  |  |  |  |  |
| Fall | 5.58 | 2.63–11.83 | <0.001^*^ |  | 3.00 | 1.31–6.86 | 0.009^*^ | 1.21 |

Abbreviations: ALT, alanine aminotransferase; AST, aspartate aminotransferase; BMI, body mass index; BUN, blood urea nitrogen; CI, confidence interval; CKD, chronic kidney disease; COPD, chronic obstructive pulmonary disease; GERD, gastroesophageal reflux disease; HR, hazard ratio; N.D., not determined; NSAIDs, non-steroidal anti-inflammatory drug; VIF, variance inflation factor. ^*^*p* < 0.05.

Table S6 Multivariate Cox regression analysis to estimate the hazard ratios for developing dementia with propensity score as a variable

| Variables | HR | 95% CI | *p*-value | VIF |
| --- | --- | --- | --- | --- |
| Delirium | 6.48 | 3.65–11.48 | <0.001^*^ | 2.32 |
| Propensity score | 0.60 | 0.24–1.46 | 0.258 | 2.32 |

Abbreviations: CI, confidence interval; HR, hazard ratio; VIF, variance inflation factor. ^*^*p* < 0.05.
